# Pulsating Evidence: Clinical Visualization of an Abdominal Aortic Aneurysm

**DOI:** 10.7759/cureus.88769

**Published:** 2025-07-25

**Authors:** Bhavya Gandhi, Morgan Rosenberg, Jane Y Wang

**Affiliations:** 1 School of Medicine, McMaster University, Hamilton, CAN; 2 Family Medicine, University of Toronto, Toronto, CAN; 3 Family Medicine (Emergency Medicine), Michael Garron Hospital, Toronto, CAN

**Keywords:** aortic abdominal aneurysm, clinical examination skills, clinical visualization, pulsatile mass, thorough physical examination

## Abstract

Abdominal aortic aneurysm is a critical medical condition typically diagnosed using imaging such as CT of the abdomen and/or ultrasonography. Physical examination is often considered unreliable for definitive diagnosis. This case report presents a unique case of a male patient in their 70s with a large, visible abdominal aortic aneurysm on inspection of the abdomen, seen as a pulsating mass. This case report highlights the relevance of physical examination to support the diagnosis and management of abdominal aortic aneurysms.

## Introduction

Abdominal aortic aneurysm (AAA) is a critical medical condition generally referring to dilation of the abdominal aorta greater than 3.0 cm, with those greater than 5.0 cm at a high risk of rupture. Males over 65 years old with a smoking history are generally at the highest risk for an AAA. While physical examination can provide some clinical evidence of an AAA, imaging or ultrasonography provides a definitive diagnosis [1]. While textbooks often describe AAAs as presenting with a pulsatile abdominal mass, sensitivity for this maneuver has been reported at only 68% with a specificity of 75%; hence, physical examination is often underutilized [1]. This report aims to underscore the diagnostic value of bedside physical examination in an era of increasing reliance on imaging while highlighting an incidental but striking clinical visualization of an AAA. We hope to renew clinical interest in maintaining strong physical examination skills, particularly in patients where imaging may be delayed or inaccessible.

## Case presentation

A frail 77-year-old male was admitted to the emergency department following a transient episode of syncope lasting approximately five seconds, accompanied by refractory hypotension. His medical history included a remote left above-knee amputation secondary to gangrenous foot and a known AAA first diagnosed four months prior during admission for *Mycobacterium avium* pneumonia. Other relevant past medical history included type 2 diabetes mellitus (on empagliflozin 10 mg once daily) and dyslipidemia (on atorvastatin 80 mg once daily). There was no history of substance use, including smoking.

**Figure 1:**
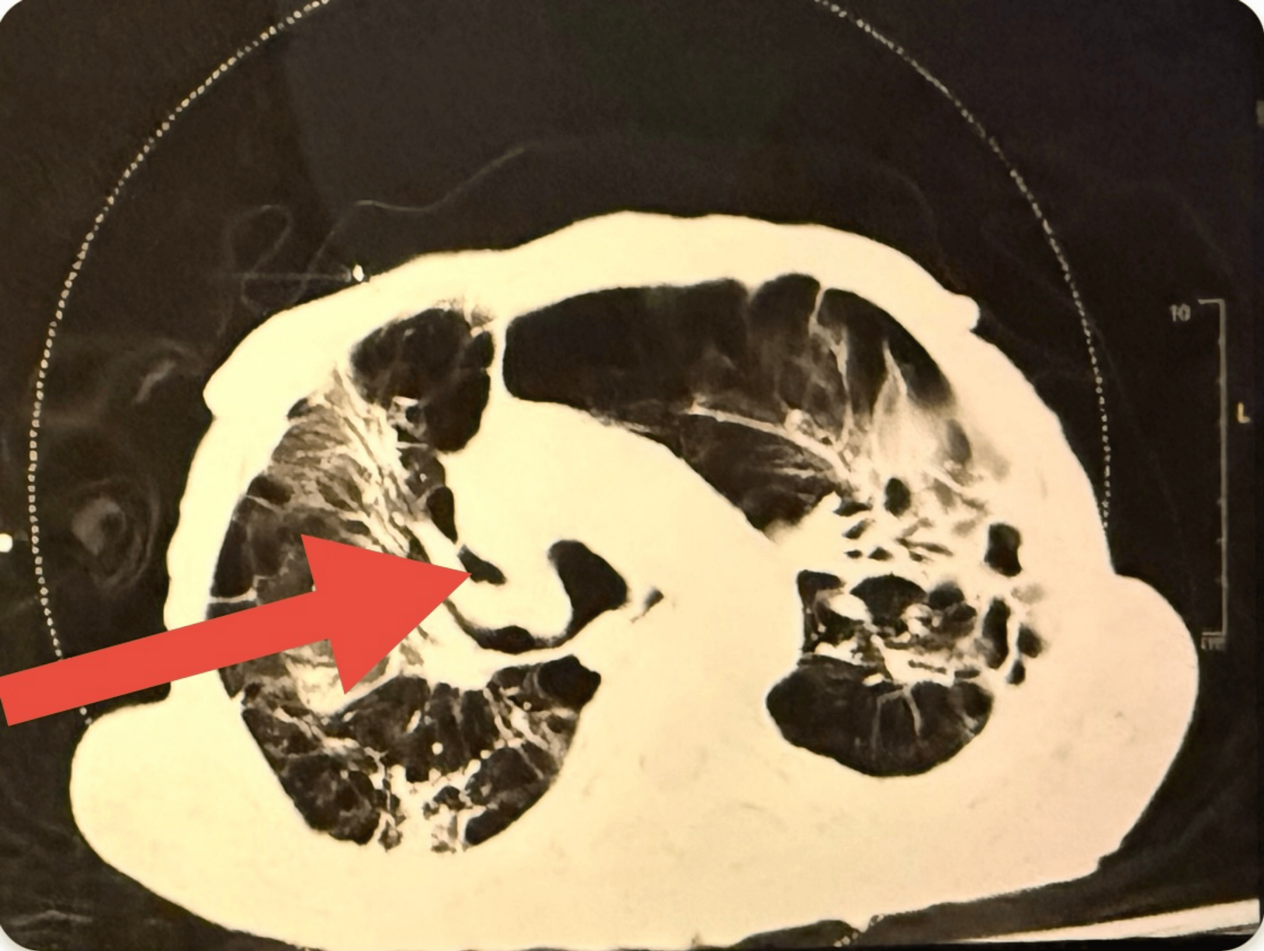
CT of the abdomen showing a 7.6 × 6.2 cm infrarenal abdominal aortic aneurysm. The red arrow indicates the abdominal aortic aneurysm.

Upon reviewing past medical history in the patient’s chart, a CT of the abdomen was ordered in the emergency department to assess for interval change and stability of the AAA, showing a 7.6 × 6.2 cm AAA (Figure 1). This was similar in dimensions to a CT from four months prior. The patient was previously seen by the vascular surgery team during his previous admission for pneumonia four months prior. Given his acute state, he was deemed nonoperative at that time and was advised to follow up in the outpatient vascular surgery clinic for consideration of elective AAA repair after clinical stabilization per the patient’s discretion and goals of care.

During this admission, the patient was asymptomatic from the AAA. His legs were warm with no concerns for acute limb ischemia. He had appropriate bilateral Doppler femoral signals with intermittent distal right Dopplers. The patient maintained his previous decision not to proceed with surgical repair of this AAA, given his goals of care, which were for non-invasive measures only.

Initial examination during this admission did not reveal any clinical signs of an AAA. However, upon secondary examination, clinical visualization of his AAA in the form of visible pulsations in the periumbilical abdominal region became apparent. Unlike the normal aortic pulsation sometimes visible in thin individuals, this patient exhibited a grossly visible, broad pulsatile mass extending across the periumbilical region, consistent with a large aneurysmal dilation (Video 1). The patient’s abdomen was entirely nontender and nondistended, including in the region of the pulsations. This visualization of the AAA can be partly attributed to frailty secondary to nutritional deficits. A nutritional assessment showed high nutritional risk due to inadequate oral intake related to changes in taste and appetite or preference, as evidenced by recent weight loss.

**Video 1:** Visualization of the patient’s abdomen. A prominent pulsatile mass is seen in the periumbilical region, consistent with a large abdominal aortic aneurysm.

## Discussion

While the patient presented with a transient syncopal episode and hypotension, the AAA was not considered the direct cause. Rather, the aneurysm was a known, stable finding that was re-evaluated during the admission. This highlights the importance of maintaining a broad diagnostic lens and performing a full physical examination, even when the presenting complaint appears unrelated.

This case illustrates the enduring clinical value of bedside examination, particularly inspection, which is often overlooked in modern practice. The visibility of the aneurysm in this patient was likely due to a combination of factors, including its large size (7.6 × 6.2 cm) and the patient’s low body mass index and recent weight loss.

Classically, physical examination, specifically abdominal inspection and palpation, is not known to have high sensitivity for AAA detection, with studies previously reporting a 68% sensitivity and 75% specificity [1]. However, this case and associated video highlight the importance of a high-quality, comprehensive physical examination in the context of AAA detection. Patients, as seen in this case, can often be asymptomatic [2]. Without diagnostic imaging, such large AAAs may easily be missed, likely leading to worsened patient outcomes [3]. As such, a physical examination, including inspection of the abdomen for any masses, lesions, or pulsations; auscultation for items such as bruits; and palpation for masses, tenderness, or pulsations, should be conducted. The most common location to palpate or visualize an AAA is the periumbilical region, though epigastric pulsations may also occur [4]. In this case, the pulsations were broad, dome-shaped, and clearly visible with the patient supine, consistent with a large AAA. Transmitted aortic pulsations in thin individuals may also be hepatic artery aneurysms or pancreatic tumors.

The educational significance of this case lies in the striking visual detectability of the aneurysm on simple examination, an uncommon but powerful clinical finding. While textbooks describe the presence of a pulsatile abdominal mass, it is observed in only a minority of patients with large AAAs. Video 1 provides a valuable teaching resource to reinforce the importance of bedside skills and the diagnostic potential of inspection.

While advanced medical modalities such as diagnostic imaging have revolutionized modern medicine, a physical examination can still yield important clinical findings. This case emphasizes the need to preserve and teach physical examination techniques, especially in environments where imaging may be delayed, unavailable, or unaffordable. In particular, clinicians should be reminded not to omit inspection of the abdomen, especially in patients at high risk for vascular pathology.

## Conclusions

This case report provides evidence for an uncommon presentation of AAA. AAAs may remain clinically stable and subjectively asymptomatic to patients despite their large size. While imaging and ultrasonography are common methods for diagnosing AAA in a hospital setting, clinicians should consider that AAA may be visualized on physical examination. A high-quality, comprehensive physical examination can yield important clinical findings pertinent to a patient’s outcomes and can potentially support rapid development and implementation of management plans.
